# CDX2 confers ferroptosis resistance in stage II-III colon cancer via upregulation of NUPR1

**DOI:** 10.1038/s41419-026-08412-x

**Published:** 2026-03-12

**Authors:** Junhui Yu, Mingchao Mu, Chenye Zhao, Xiaopeng Li, Xueqian Ma, Zepeng Dong, Xuejun Sun, Jianbao Zheng

**Affiliations:** Department of General Surgery, the First Affiliated Hospital of Xi’an Jiaotong University, Xi’an, Province PR China

**Keywords:** Cancer therapeutic resistance, Predictive markers

## Abstract

High CDX2 expression frequently indicates better survival in stage II-III colon cancer; nevertheless, it is linked to decreased systemic chemotherapy response rates. Ferroptosis, commonly recognized as an iron-dependent oxidative death, is increasingly believed as a disease-modifying mechanism. The purpose of this study is to determine the role of ferroptosis in CDX2-mediated colon cancer chemical resistance. Mechanistically, CDX2-mediated NUPR1 transcription prevents ferroptotic cell death by reducing iron accumulation and oxidative stress damage. Depletion of NUPR1 counteracted the effect of CDX2 overexpression in terms of ferroptosis resistance, whereas transfection-enforced re-expression of NUPR1 restores ferroptosis resistance in CDX2-deficient cells. Genetic or pharmacological blockage of CDX2-NUPR1 axis improved the potential of ferroptosis agonists to combat colon cancer in preclinical mouse models. Our study uncovered a novel molecular mechanism by which CDX2 imparts ferroptosis resistance to colon cancer. Blockage of NUPR1 might be as a potential therapeutic strategy for CDX2-positive stage II-III colon cancer.

**Figure:**
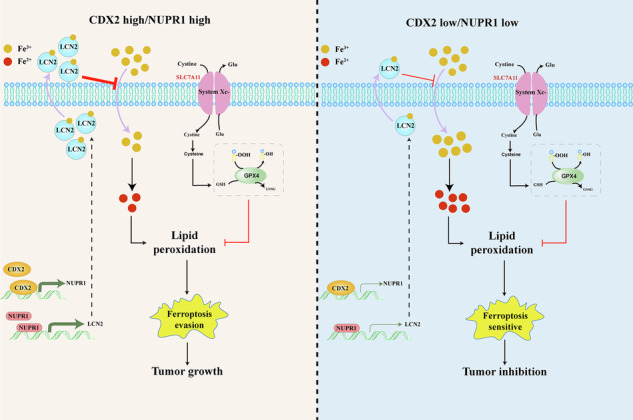
The current study demonstrated a positive correlation between CDX2 expression and chemical resistance in colon cancer. Mechanistically, CDX2 directly transactivates NUPR1 and subsequent its target LCN2 to confer ferroptosis resistance by inhibiting iron-induced oxidative damage. Genetic or pharmacological blockage of CDX2-NUPR1 axis may strengthen the anticancer efficacy of adjuvant chemotherapy on stage II-III CDX2-positive colon cancer in vitro and in vivo. Our study uncovered a novel molecular mechanism by which CDX2 confers ferroptosis resistance in colon cancer. Blockage of NUPR1 might be as a potential therapeutic strategy for stage II-III CDX2-positive colon cancer.

The current study demonstrated a positive correlation between CDX2 expression and chemical resistance in colon cancer. Mechanistically, CDX2 directly transactivates NUPR1 and subsequent its target LCN2 to confer ferroptosis resistance by inhibiting iron-induced oxidative damage. Genetic or pharmacological blockage of CDX2-NUPR1 axis may strengthen the anticancer efficacy of adjuvant chemotherapy on stage II-III CDX2-positive colon cancer in vitro and in vivo. Our study uncovered a novel molecular mechanism by which CDX2 confers ferroptosis resistance in colon cancer. Blockage of NUPR1 might be as a potential therapeutic strategy for stage II-III CDX2-positive colon cancer.

## Introducation

Colorectal cancer (CRC) is the second most frequently diagnosed malignancies worldwide [1]. Adjuvant chemotherapy offered after colon cancer surgery reduced the relapse risk of patients with stage III and high-risk stage II [2]. The current definition of high-risk II stage colon cancer was still in dispute [3]. Since a considerable proportion of patients with stage II have a favorable prognosis, the hazards of chemotherapy must be balanced against the benefits of survival. Hence, searching for the robust biomarkers identifying patients responding well to adjuvant chemotherapy might greatly improve treatment stratification across tumor stages.

The caudal type homeobox transcription factor 2 (CDX2) is identified as a master regulator of intestinal cell survival and differentiation [4]. CDX2 is normally restricted to the intestinal epithelium, where it is abundant. However, in a portion of CRC, the loss of CDX2 is closely related to aggressive clinicopathological profile and poor survival [5, 6]. Mechanistically, reduced CDX2 in triggering colorectal tumorigenesis is a very complex process involving different mechanism including NF-κB, Wnt/β-catenin, or PI3K/Akt via functioning as a transcriptional factor. In contrast to its defined role as a tumor suppressor, CDX2 when amplified is essential for colorectal cancer cell proliferation and survival [7]. Hence, CDX2 might have dual roles as an oncogene and tumor suppressor in certain context. A landmark study by Dalerba et al. revealed that the absence of CDX2 expression defined a high-risk subset of patients with localized colon cancer and was roughly linked to poor survival in stage II-III CRC [8]. Hence, postoperative adjuvant chemotherapy might be recommended for patients with stage II CDX2-negative colon cancer. Intriguingly, CDX2-negative patients benefited from adjuvant treatment more than CDX2-positive patients did [8], implying that CDX2 deficiency might confer chemotherapy sensitivity. Nevertheless, the potential mechanism underlying chemotherapy sensitivity conferred by CDX2 depletion remains unclear and requires further investigation.

Ferroptosis is a recently discovered iron-dependent cell death caused by an abnormal accumulation of lipid hydroperoxides and membrane damage [9]. Ferroptosis has piqued the interest of researchers due to potential therapeutic benefits in a variety of human diseases including cancers by specifically targeting ferroptosis [10–14]. However, tumors are vulnerable to developing ferroptosis resistance through a variety of mechanisms, including reduced polyunsaturated lipid levels and increased antioxidant capability [15]. Escaping from ferroptosis may severely lower the effectiveness of chemotherapeutics and almost certainly result in drug resistance and cancer recurrence [16]. Therefore, it is vitally necessary to identify the precise mechanisms underlying ferroptosis resistance and, using those insights, to develop a therapeutic approach to resensitize tumor cells to ferroptosis.

Nuclear protein 1 (NUPR1) is an AT hook-containing chromosomal DNA-binding protein that was first discovered and cloned in pancreatitis-induced tissue injury [17]. Growing evidence indicated that NUPR1 responds to a variety of environmental stresses, such as oxidative damage and the unfolded protein response (UPR) [18–20]. Ablation of NUPR1 in tumor cells causes mitochondrial dysfunction and energy metabolism, raises ROS levels, and initiates cellular death processes such as apoptosis, autophagy, necroptosis, and ferroptosis [19, 21–23]. NUPR1 expression and activity are tightly controlled at multilevel mechanism including transcription, epigenetic modification, and posttranslational regulation [24–26]. The precise regulatory mechanism of NUPR1 under tumor chemical resistance has yet to be fully clarified.

In this study, we intent to evaluate the correlation between CDX2 expression and its target gene involved in ferroptotic cell death during chemical resistance in stage II-III colon cancer.

## Materials and methods

### Cell cultures

Human colon cancer cells were purchased from Cell Bank, Chinese Academy of Sciences (Shanghai, China). HT-29, SW480, and Caco-2 cells were all cultured in DMEM supplemented with 10% fetal bovine serum at 5% CO2 at 37 °C. Once the cells reached 70% confluence, they were subjected to further research.

### Lentiviral vectors and transfection

Lentiviral vectors with CDX2/NUPR1 shRNA or cDNA were purchased from GeneChem Co., Ltd. (Shanghai, China). All transfections were performed according to the manufacturer’s instructions.

### Transmission electron microscopy (TEM)

Briefly, cells were fixed with electron microscope fixing solution for 2–4 h. Then cells were embedded with 1% agarose, dehydrated, and cut to ultrathin sections (60–80 nm) with ultramicrotome (Leica UC7, Leica). Sections were stained with uranyl acetate and lead citrate in a Leica EM Stainer, and examined using a JEM 1010 transmission electron microscope (JEOL, USA, Inc., Peabody, MA) at an accelerating voltage of 80 kV.

### CCK8, colony formation, and apoptosis assays

CCK8 assays were performed as described previously [27]. For colony formation assay, three hundred cells were seeded and cultured for 14 days. Colonies (≥ 50 cells/colony) were counted. For apoptosis assay, cells were labeled with Annexin V PE/7-AAD (BD Biosciences, Franklin Lakes, NJ, USA) according to the manufacturer’s protocol as previously described [28]. Each experiment was performed in triplicate.

### Nude mouse xenograft assay

The experiment was approved by the First Affiliated Hospital of Xi’an Jiaotong University Experimental Animal Ethics Committee. For each model, we subcutaneously transplanted × 10^6^ cells with 0.1 ml PBS into the right dorsal flanks of 6-week-old female nude mice. Eight days post-inoculation, the tumor-bearing mice were separated randomly into groups of five mice each. The treatment was initiated by drug intraperitoneal administration every three days. Mice were euthanized after 24 days post-inoculation. Tumors were measured with a caliper every four days and volume was calculated as ½(length × width^2^). At the end of the experiment, the mice were sacrificed and the xenograft tumors were isolated and weighted.

### RNA isolation and real-time PCR

RNA isolation, complementary DNA (cDNA) synthesized and real-time PCR were performed as described previously [27]. The sequences of primers were summarized in Supplementary Table 1. Each experiment was performed in triplicate.

### Microarray analysis

Microarray analysis was carried out to compare gene expression profile in SW480-shCDX2 and the control cells. Total RNA was extracted with TRIzol reagent and prepared for subsequent analysis by Affymetrix GeneChip system (Genechem Co., Ltd).

### Immunohistochemistry (IHC)

The IHC staining was carried out as described previously [27]. Briefly, the extent of stained cells (0, 0–5%; 1, 6–25%; 2, 26–50%; 3, 51–75%; 4, 76–100%) and the staining intensity (0, negative; 1, light brown; 2, brown; 3, dark brown) were recorded. The immunoreactivity scores (IRSs) were defined as the product of extent and intensity scores. An IRS of >3 was considered as positive expression.

### Total Protein extraction and Western blot

The elaborate protocol was carried out exactly as described previously [27]. The antibody data was provided in Supplementary Table 2. Each experiment was performed in triplicate.

### Luciferase reporter assay

For promoter analyses, we designed a NUPR1 full promoter reporter construct (from-2931 bp to +174 bp) and the truncated ones (Supplementary Table 1). The plasmids containing firefly luciferase reporters of NUPR1 promoter and the truncated ones and the pTK-RL plasmids were co-transfected into cells. The detailed protocol was carried out as described previously [27].

### Patient-derived xenograft (PDX) model

Tumor tissues were obtained from patients with CRC after surgical resection at the First Hospital of Xi’an Jiaotong University and placed on iced DMEM supplemented with 10% FBS. The tumor tissues were chopped into 1–2 mm^3^ small pieces and subcutaneously implanted into the dorsal flank of 6-week-old male NOD/SCID mice. The tissues from mice bearing PDX tumors were harvested and cut into pieces when the tumor volume reached 1–2 cm^3^, and then implanted into BALB/c nude mice. Every three days, callipers were used to measure the size of the tumors. Using the equation volume = ½(length × width^2^), the volume of tumors was determined. Mice were divided into four treatment groups at random when tumor sizes reached 50 mm^3^. After receiving medication treatment for four weeks, mice were euthanized, and the xenograft tumors were isolated and weighted. The tumors were harvested for further analysis.

### Measurement of lipid ROS

To determine Lipid ROS, cells were pretreated with drugs followed by incubation with C11 BODIPY 581/591 (50 μM) for 1 h. Cells were washed twice with PBS to remove excess dye. Cells were then digested with trypsin and resuspended in PBS containing 5% FBS, and was analyzed by flow cytometry.

### Lipid peroxidation assay

MDA lipid peroxidation assay kit (ab118970, Abcam, Cambridge, UK) was used according to the manufacturer’s specifications.

### Measurement of 8-OHdG

The quantitative determination of 8-OHdG is detected by 8-OHdG ELISA Kit (ab285254, Abcam, Cambridge, UK) according to the manufacturer’s instructions.

### Iron level detection

The intracellular iron concentration was measured using the iron assay kit (ab83366; Abcam, Cambridge, UK) following the manufacturing instructions.

### Quantitative chromatin immunoprecipitation (qChIP)

The EZ-ChIP Kit (Millipore, Bedford, MA, USA) was utilized to perform qChIP. The elaborate protocol was carried out as previously described [27]. Using special primers, real-time PCR was employed to amplify the regions of the CDX2 binding site of NUPR1 promoter (Supplementary Table 1). Each experiment was performed in triplicate.

### Statistical analysis

The Student’s t-test or one-way ANOVA were utilized to compare the differences among the groups. The SPSS version 22.0 (SPSS Inc., Chicago, IL, USA) was employed for all statistical calculations. Statistical significance was set at *P* < 0.05.

## Results

### CDX2 promoted the chemical resistance of colon cancer

**Fig. 1:**
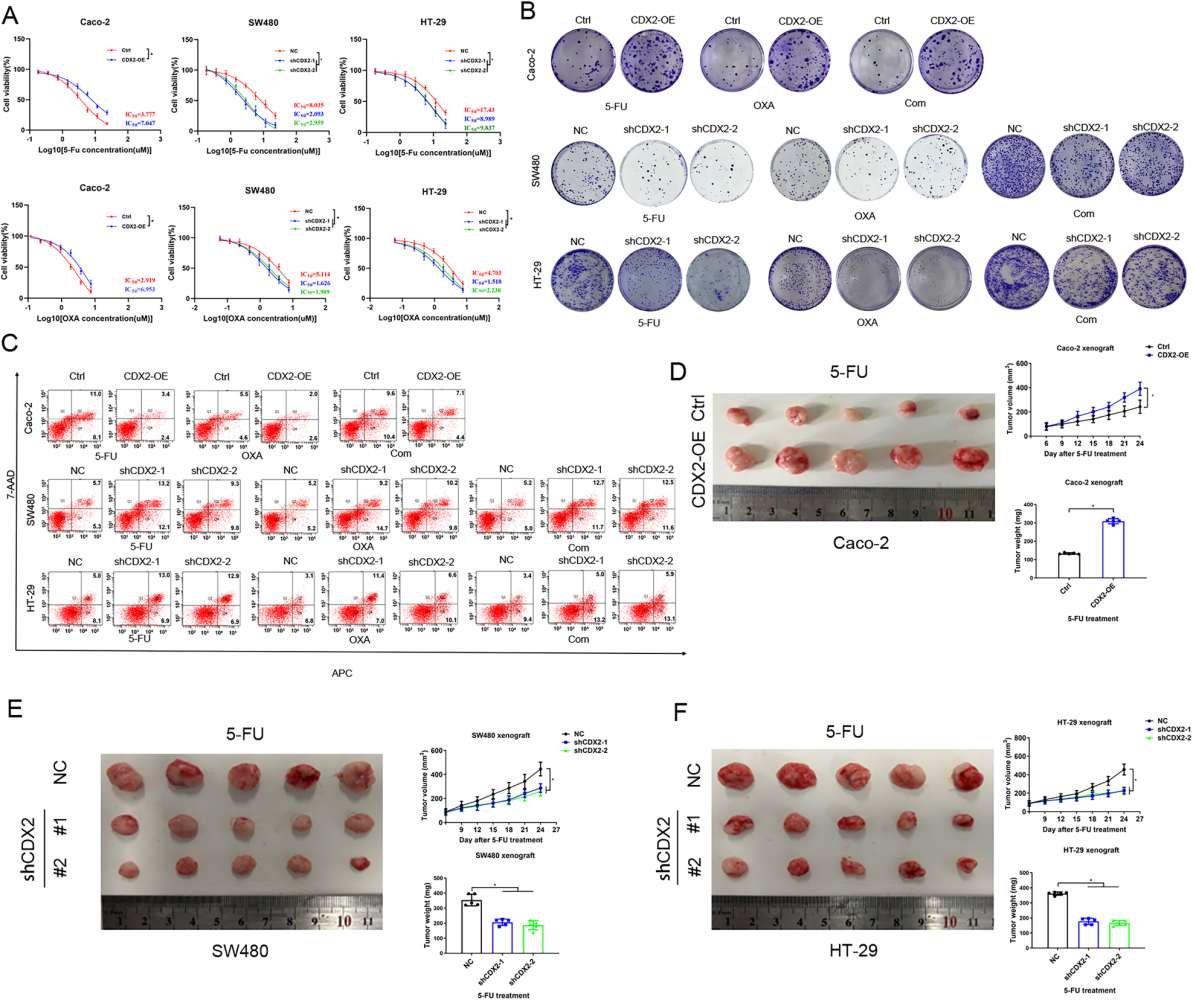
CDX2 promoted the chemical resistance of colon cancer. **A**–**C** The effect of CDX2 depletion or overexpression on cell viability (**A**), colony formation (**B**), and apoptosis (**C**) of colon cancer cells to 5-FU or oxaliplatin alone or in combination. **D** Schematic representation, tumor growth curves and tumor weights of the tumor xenografts formed by CDX2-overexpressing Caco-2 cells and the control cells treated with 5-FU. **E**, **F** Schematic representation, tumor growth curves and tumor weights of the tumor xenografts formed by CDX2-depleted SW480 (**E**) and HT-29 (**F**) cells and the control cells treated with 5-FU. All data are the mean ± SD of three independent experiments. *P < 0.05.

To verify the hypothesis that CDX2 expression confers increased chemical resistance on colon cancer, we utilized CCK8 assays on colon cancer cells and discovered that Caco-2 cells ectopically expressing CDX2 were profoundly more tolerant to 5-FU or oxaliplatin alone (Fig. 1A). Depletion of CDX2, to the contrary, enhanced chemotherapy vulnerability in SW480 and HT-29 cells (Fig. 1A). The colony formation assay showed that transfection-enforced re-expression of CDX2 enhanced colony-forming ability of Caco-2 cells with 5-FU or oxaliplatin alone or in combination (Fig. 1B; supplementary fig. 1a). The reversed alteration was achieved in chemotherapy-treated HT-29 and SW480 cells upon CDX2 depletion (Fig. 1B; Supplementary fig. 1b, c). We then utilized flow cytometry to detect apoptotic and necrotic cells with Annexin V-PE/7-AAD dual staining. Chemotherapy alone or in combination-treated Caco-2 cells showed a substantial decrease in cell mortality in response to CDX2 overexpression (Fig. 1C; supplementary fig. 1d). On the opposite, CDX2 depletion showed an increase in cell death in HT-29 and SW480 cells (Fig. 1C; Supplementary fig. 1e, f). Finally, xenograft tumor models showed that upon 5-FU exposure, the xenograft tumors produced by CDX2-overexpressing cells developed more slowly and were lighter, in comparison to those created by control cells (Fig. 1D). Conversely, CDX2-depleted tumor xenograft displayed the reversed alterations (Fig. 1E, F). In summary, the evidence indicated that CDX2 expression positively correlates with chemical resistance of colon cancer.

### NUPR1 expression is positively correlated with CDX2 in Stage II-III Colon Cancer

**Fig. 2:**
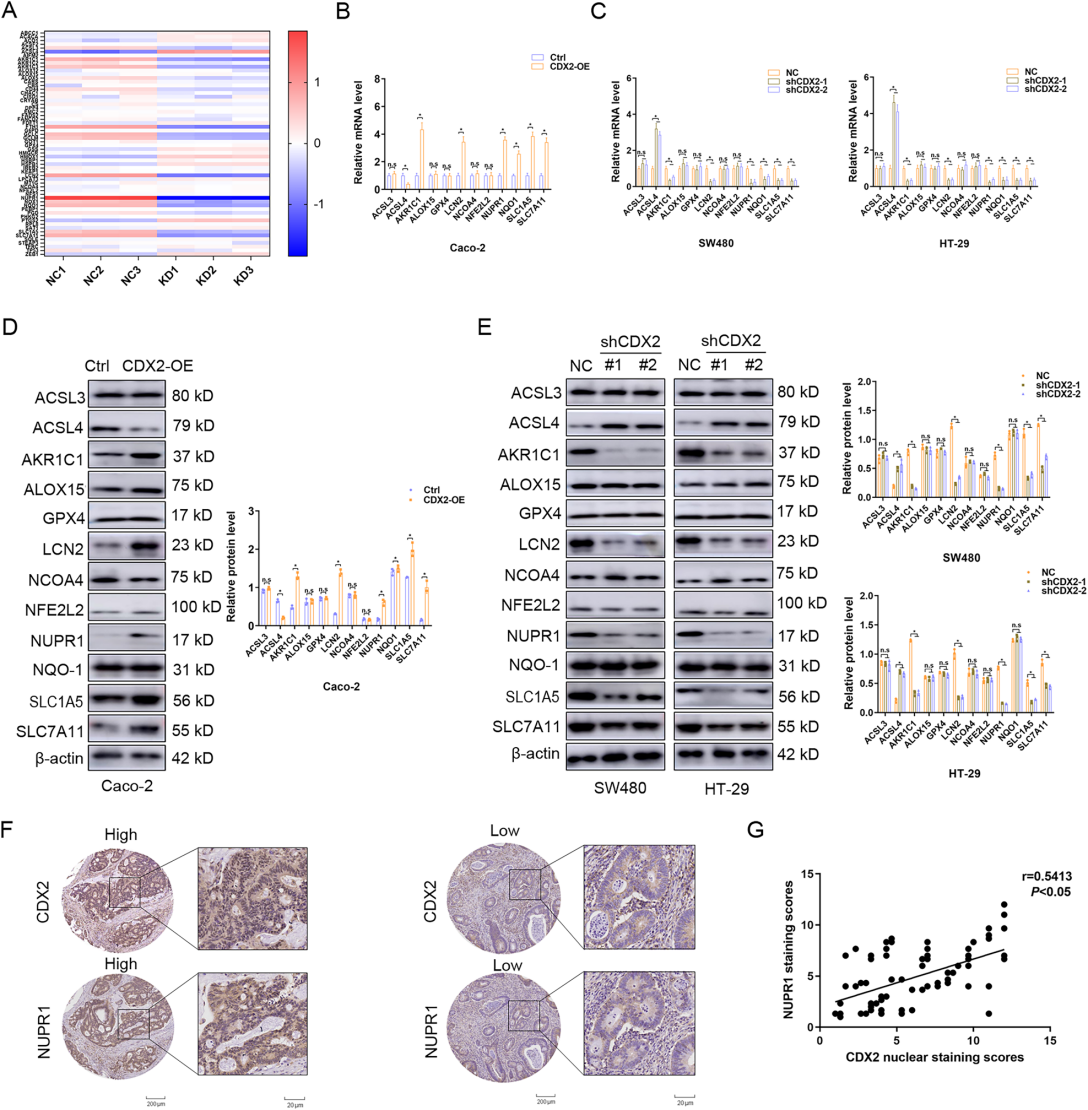
NUPR1 expression is positively correlated with CDX2 in Stage II-III Colon Cancer. **A** Visualization of differentially ferroptosis-associated genes engaged for iron metabolism, iron absorption, and cystine metabolism of of Gene Ontology Enrichment analysis in SW480-shCDX2 and the control cells. **B**, **D** The mRNA (**B**) and protein (**D**) level of several ferroptosis-associated genes in CDX2-overexpressing Caco-2 cells detected by real-time PCR or western blotting analysis. **C**, **E** The mRNA (**C**) and protein (**E**) levels of ferroptosis-associated genes in CDX2-depleted SW480 and HT-29 cells detected by real-time PCR or western blotting analysis. **F** CDX2 and NUPR1 expression in tissue microarrays purchased from Shaanxi Kexin Biotechnology Co., Ltd, including 90 pairs of CRC samples and paired NC tissues by IHC staining (cohort Ⅰ). **G** Correlation analysis of CDX2 and NUPR1 in CRC tissue microarrays. All data are presented as the mean ± SD from three independent experiments. *P < 0.05.

Using microarray analysis on SW480-shCDX2 and control cells, the underlying mechanisms of CDX2 on CRC chemical resistance was explored. Several ferroptosis-associated genes engaged in iron metabolism, iron absorption, and cystine metabolism were discovered through GO enrichment analysis (Fig. 2A). These data greatly inspire us that ferroptosis might be implicated in CDX2-medicated chemical resistance of colon cancer. Afterward, the impact of CDX2 modification on the expression of crucial genes involved in ferroptosis was evaluated. As expected, we found that ectopic CDX2 expression was sufficient to promote endogenous AKR1C1, LCN2, NQO-1, NUPR1, SLC1A5, and SLC7A11 expression and inhibit ACSL4 expression at both mRNA and protein levels, but did not affect other genes such as ACSL3, ALOX15, GPX4, NCOA4, or NFE2L2 (Fig. 2B, D). Depletion of endogenous CDX2 achieved the reversed alterations in HT-29 and SW480 cells (Fig. 2C, E).

The clinical association between CDX2 and NUPR1 in CRC was investigated by using IHC staining on tissue microarrays containing 90 stage II-III colon cancer tissues (cohort I). In colon cancer tissues, NUPR1 protein was primarily found in the nucleus (Fig. 2F). When compared to CDX2-low tumors, NUPR1 protein expression was shown to be considerably higher in CDX2-high tumors (Fig. 2F). Moreover, Spearman correlation analysis indicated that NUPR1 protein levels were consistently associated favorably with CDX2 protein levels in stage II-III colon cancer tissues (Fig. 2G). Another independent cohort II of 228 stage II-III colon cancer tissues supported a positive relation between CDX2 and NUPR1 (Supplementary fig. 2a–c). These data collectively suggest that CDX2 positively correlates with NUPR1 in Stage II and Stage III colon cancer.

### CDX2 transcriptionally upregulates NUPR1 expression

**Fig. 3:**
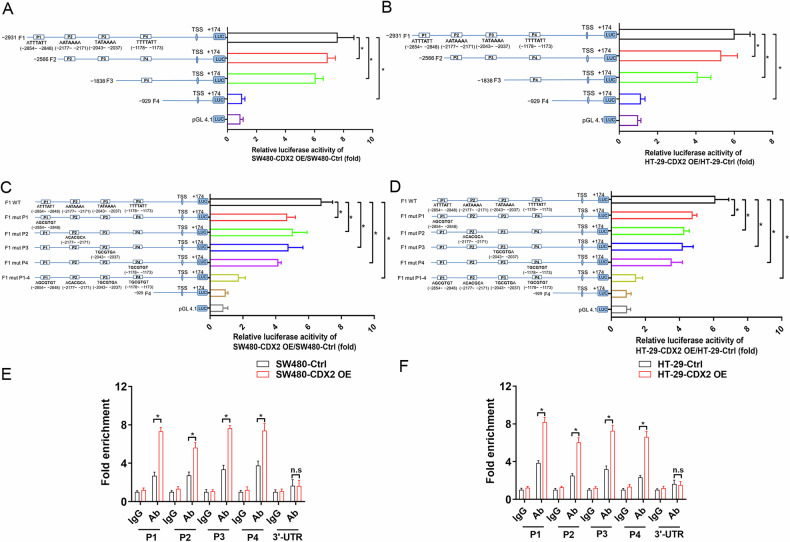
CDX2 transactivates the expression of NUPR1 by binding to the NUPR1 promoter in colon cancer cells. **A**, **B** The activities of the NUPR1 full promoter reporter construct and the truncated ones in CDX2-overexpression SW480 (**A**) and HT-29 (**B**) cells using the dual-luciferase assay. **C**, **D** The effect of the single and multiple mutations of the putative CDX2-binding sites (P1-4) on the activities of the NUPR1 full promoter reporter construct in CDX2-overexpression SW480 (**c**) and HT-29 (**D**) cells using the dual-luciferase assay. **E**, **F** Enrichment level of the CDX2- binding putative site in the NUPR1 promoter region in SW480 (**E**) and HT-29 (**F**) cells determined by the qChIP assay. All data are presented as the mean ± SD from three independent experiments. *P < 0.05.

The clinical relevance of CDX2 and NURP1 in Stage II and Stage III colon cancer prompted us to investigate whether NUPR1 was transcriptionally regulated by CDX2, considered that CDX2 is an intestine-special transcription factor. We utilized JASPER algorithm to search potential CDX2-binding sites in the NUPR1 promoter region relative to the transcription start site (TSS). The 3.0 kb region upstream of the TSS contained four presumptive CDX2-binding sites (Fig. 3A, B): P1 (5’-A**TTTAT**T-3’, from −2,854 bp to −2,848 bp), P2 (5’-A**ATAAA**A-3’, from −2,177 bp to −2,171 bp), P3 (5’-T**ATAAA**A-3’, from −2,043 bp to−2,037 bp), and P4 (5’-T**TTTAT**T-3’, from −1178 bp to −1173 bp). Several reporter gene constructs were created in order to evaluate the function of the alleged CDX2-binding sites affect the regulation of NUPR1 transcription. In comparison to control cells, CDX2-overexpressing cells showed considerable activity for reporter gene constructs including 3.0 kb of a 5′-flanking region (F1, −2931/ + 174 bp) from the NUPR1 gene (Fig. 3A, B). However, transfection of the truncated fragment F2 (P1 depletion, −2566/ + 174 bp) or F3 (P1-3 depletion, −1838/ + 174 bp) gradually lowered the luciferase activity (Fig. 3A, B). We observed the lowest activity of NUPR1 reporter gene constructs with deletions upstream of the −1.0 kb pair location (F4, −929/ + 174 bp) in CDX2-overexpressing cells, implying that sequences between −3.0 kb and −1.0 kb pairs are crucial for triggering NUPR1 transcription. Furthermore, the putative CDX2-binding sites (P1-4) in this region are all critical for the activation of NUPR1 transcription, as evidenced by analysis of single and multiple mutations in these sites (Fig. 3C, D).

The direct in vivo binding of CDX2 to the NUPR1 promoter was also examined by qPCR-ChIP experiment. In CDX2-overexpressing cells, our study found an improvement in CDX2 binding to the P1-4 segments of the NUPR1 promoter compared to control cells (Fig. 3E, F). These findings altogether indicated that CDX2 might bind to the P1-4 segment of the NURP1 promoter and trigger NUPR1 transcription in colon cancer cells.

### CDX2 protects colon cancer cells from ferroptosis through the inhibition of iron-dependent oxidative damage

**Fig. 4:**
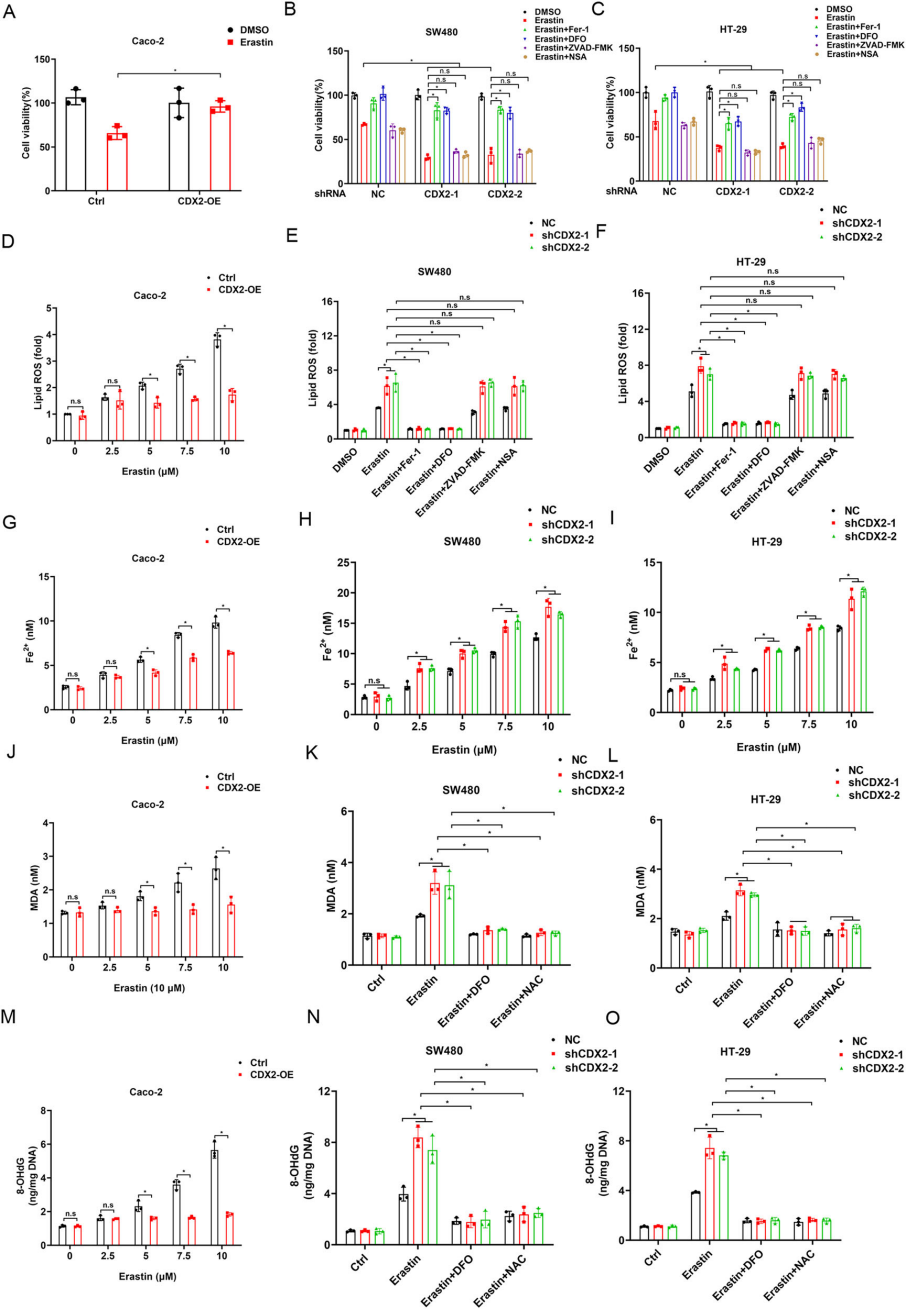
CDX2 protects colon cancer cells from ferroptosis through the inhibition of iron-dependent oxidative damage. **A**, **D**, **G**, **J**, **M** Cell viability (**A**), lipid ROS (**D**), intracellular Fe^2+^ (**G**), intracellular MDA (**J**), and intracellular 8-OHdG (**M**) in Caco-2 cells with CDX2 overexpression following treatment with erastin. **B**, **E**, **H**, **K**, **N** Cell viability (**b**), lipid ROS (**E**), intracellular Fe^2+^ (**H**), intracellular MDA (**K**), and intracellular 8-OHdG (**N**) in SW480 cells with CDX2 knockdown following treatment with erastin in the absence or presence of Fer-1, DFO, ZVAD-FMK, or NSA. **C**, **F**, **I**, **L**, **O** Cell viability (**C**), lipid ROS (**F**), intracellular Fe^2+^ (**I**), intracellular MDA (**L**), and intracellular 8-OHdG (**O**) in HT-29 cells with CDX2 knockdown following treatment with erastin in the absence or presence of Fer-1, DFO, ZVAD-FMK, or NSA. All data are presented as the mean ± SD from three independent experiments. *P < 0.05.

Prior research suggested that NUPR1 could prevent iron from causing oxidative damage and increase resistance to ferroptosis [23]. In the current study, we examined whether CDX2 regulates ferroptosis via NUPR1. We firstly assessed cell viability of CDX2-modified cells in response to ferroptosis activators (erastin or RSL3). Ectopic CDX2 expression eliminated growth inhibition caused by erastin or RSL3 in Caco-2 cells (Fig. 4A; supplementary fig. 3a). In HT-29 and SW480 cells, CDX2 deletion increased the vulnerability to ferroptosis; and this effect could be entirely reversed by ferroptosis inhibitors, such as ferrostatin-1 (Fer-1) and DFO, but not by inhibitors of apoptosis (such as ZVAD-FMK) or necroptosis (such as necrosulfonamide, NSA) (Fig. 4B, C; supplementary fig. 3b, c). The elevation of ROS production triggered by erastin or RSL3 in Caco-2 cells was abolished by ectopic CDX2 expression (Fig. 4D, G; supplementary fig. 3d, g). In HT-29 and SW480 cells, CDX2 deletion exacerbates erastin or RSL3-induced ROS production; and this effect could be entirely reversed by Fer-1 and DFO, but not by ZVAD-FMK or NSA (Fig. 4E, F, H, I; supplementary fig. 3e, f, h, i).

Intracellular iron levels are tightly controlled by its uptake, storage, release, and metabolism [29]. NUPR1 blocks ferroptosis via preventing intracellular iron accumulation [23]. The effect of altering CDX2 expression on intracellular iron levels was then investigated. As anticipated, after exposure to erastin or RSL3, CDX2-overexpressing HT-29 and SW480 cells had lower intracellular Fe^2+^ levels than the control cells (Fig. 4G; supplementary fig. 3g); however, the inverse shift occurred in CDX2-depleted Caco-2 cells (Fig. 4H, L; supplementary fig. 3h, I).

The increased oxidative stress brought on by iron excess may trigger ferroptosis by attacking DNA or membrane lipids [30]. By quantifying MDA and 8-OHdG, respectively, ectopic CDX2 expression prevented lipid peroxidation caused by erastin or RSL3 as well as oxidative DNA damage in Caco-2 cells (Fig. 4J, M; supplementary fig. 3j, m). HT-29 and SW480 cells that were deprived of CDX2, in contrast, displayed excessive lipid peroxidation and oxidative damage (Fig. 4K, L, N, O; supplementary fig. 3k, l, n, o). DFO and N-acetylcysteine (NAC), an antioxidant, inhibited erastin- or RSL3-induced cell death in CDX2-deficient colon cancer cells (Fig. 4K, L, N, O; supplementary fig. 3k, l, n, o), which was associated with decreased MDA and 8-OHdG level. These findings imply that CDX2 hinders ferroptosis by repressing iron-dependent oxidative damage.

### NUPR1 acts as an effector gene of CDX2 in blocking ferroptosis

**Fig. 5:**
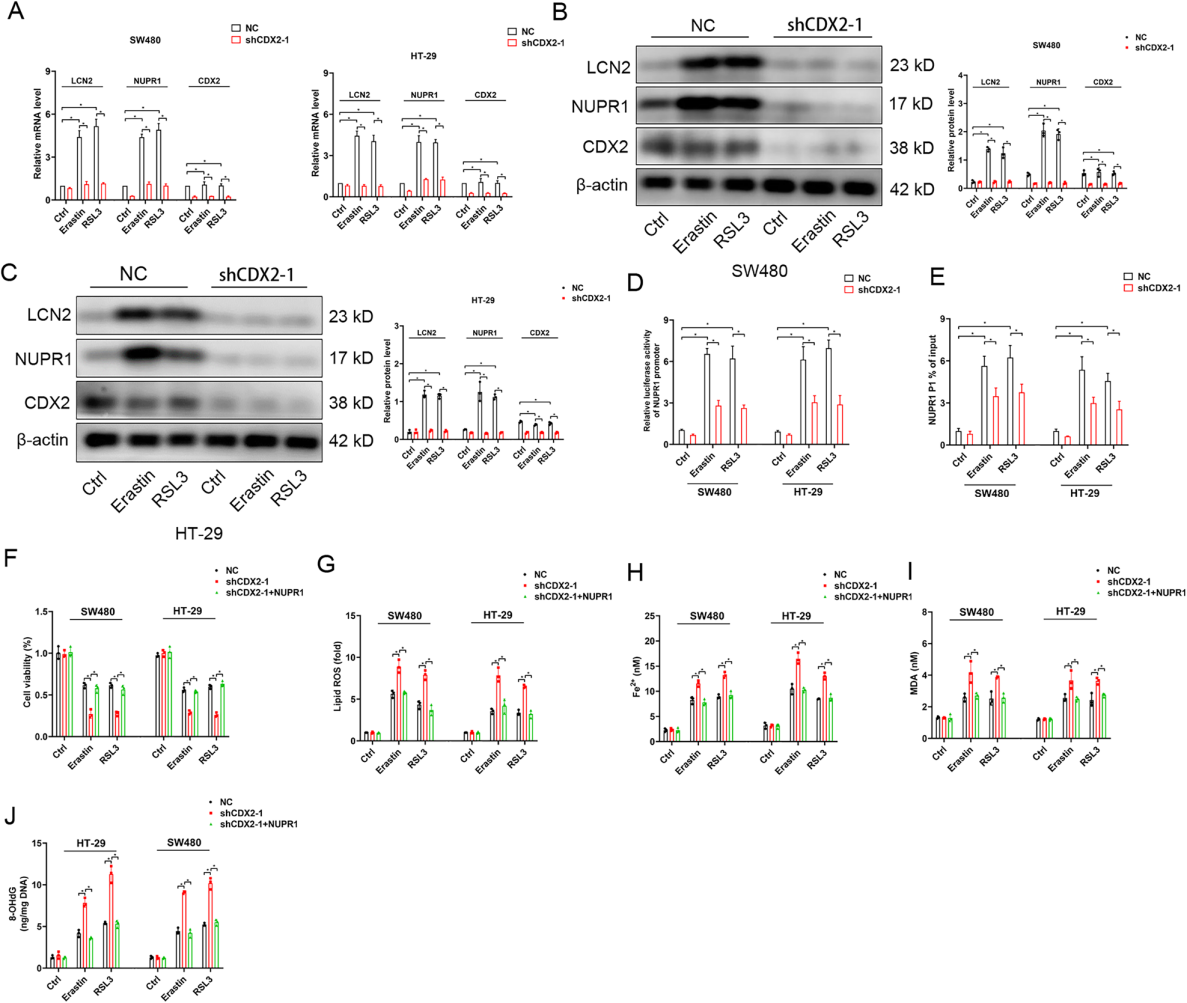
NUPR1 acts as an effector gene of CDX2 in blocking ferroptosis. **A** The mRNA levels of CDX2, NUPR1 and LCN2 in CDX2-depleted SW480 and HT-29 cells exposed to erastin and RSL3 detected by real-time PCR. **B**, **C** The protein levels of CDX2, NUPR1, and LCN2 in CDX2-depleted SW480 (**B**) and HT-29 (**C**) cells exposed to erastin and RSL3 detected by western blotting analysis. **d** The activities of the NUPR1 full promoter reporter construct in CDX2-depleted SW480 and HT-29 cells exposed to erastin and RSL3 using the dual-luciferase assay. **E** Enrichment level of the CDX2- binding putative site (P1) in the NUPR1 promoter region in CDX2-depleted SW480 and HT-29 cells exposed to erastin and RSL3 determined by the qChIP assay. **F**–**J** The effect of enhancing NUPR1 expression on cell viability (**F**), lipid ROS (**G**), intracellular Fe^2+^ (**H**), intracellular MDA (**I**), and intracellular 8-OHdG (**J**) of CDX2-depleted SW480 and HT-29 cells exposed to erastin and RSL3. All data are presented as the mean ± SD from three independent experiments. *P < 0.05.

We next attempt to investigate whether NUPR1 is implicated in ferroptosis resistance induced by CDX2. First, the elevation of NUPR1 mRNA and protein in HT-29 and SW480 cells with CDX2 abundance upon erastin or RSL3 exposure was verified by qRT-PCR and Western blotting analyses (Fig. 5A–C). LCN2, as the direct target gene of NUPR1, was regularly induced (Fig. 5A–C). However, following CDX2 depletion, erastin- or RSL3-induced elevation of NUPR1 and LCN2 was totally blocked (Fig. 5A–C). Caco-2 cells lack of CDX2 expression failed to induce NUPR1 and LCN2 expression in response to erastin or RSL3 (supplementary fig. 4a, b); while enhancing CDX2 expression elicited this induction (supplementary fig. 4a, b). As evidenced by luciferase reporter gene and qPCR-ChIP assays, NUPR1 functions as an effector gene of CDX2 in colon cancer cells (Fig. 5D, E; supplementary fig. 4c, d).

We then silenced NUPR1 in CDX2-overexpressing cells or enforcing NUPR1 expression in CDX2-depleted cells. As anticipated, deprivation of NUPR1 considerably increased the susceptibility of Caco-2 cells to ferroptosis, which was characterized by a rise in cell death (Fig. 5F), the generation of ROS (Fig. 5G), the accumulation of Fe^2+^ (Fig. 5H), and oxidative damage (Fig. 5I, J). On the opposite, in CDX2-depleted HT-29 and SW480 cells, transfected induced expression of NUPR1 recovered ferroptosis resistance (supplementary fig. 4e–i). Together, these findings show that CDX2 induces NUPR1 expression to shield colon cancer cells from ferroptosis.

### The CDX2-NUPR1 pathway limits the anticancer activity of ferroptosis inducer IKE in vivo

**Fig. 6:**
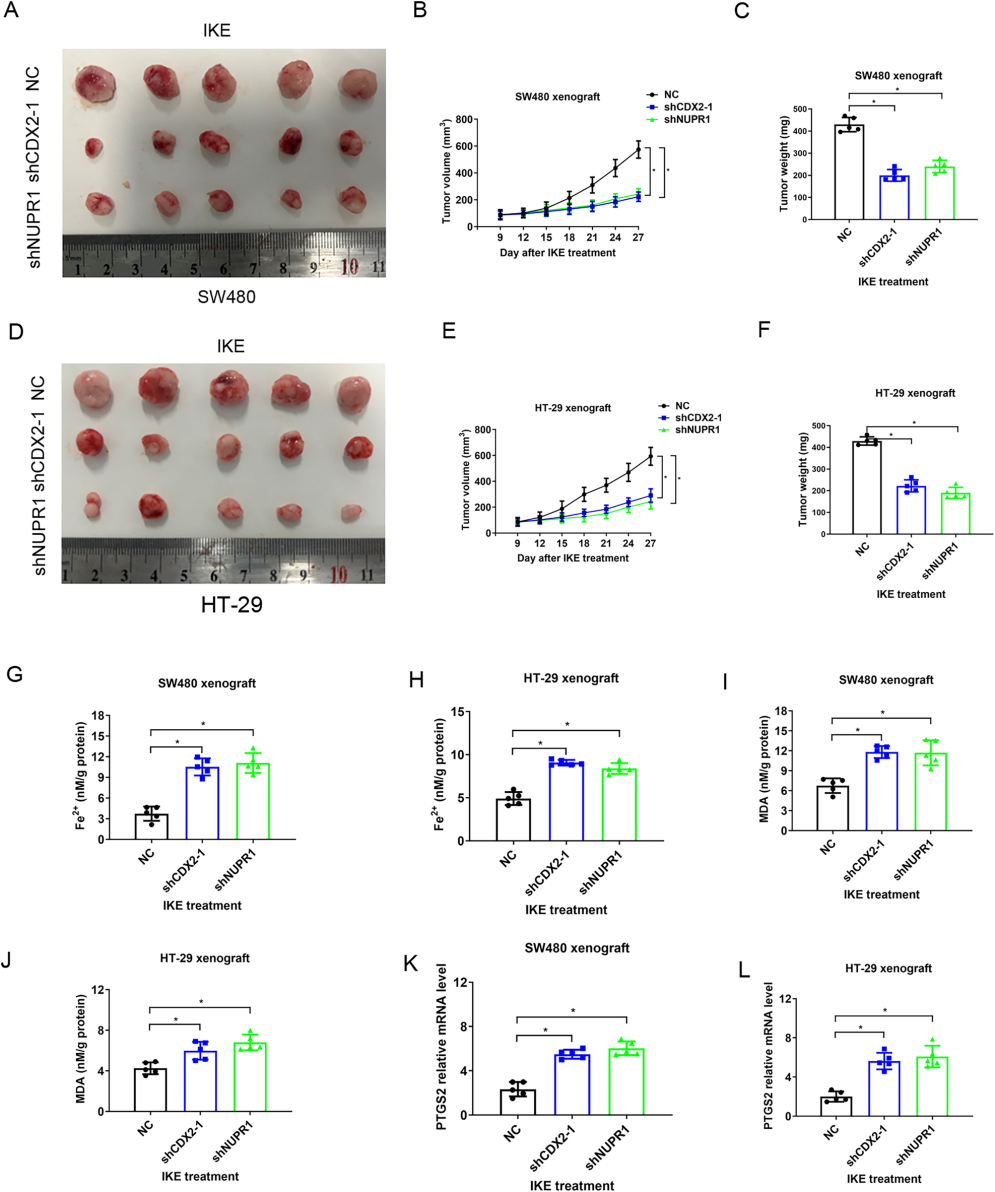
The CDX2-NUPR1 pathway limits the anticancer activity of IKE in vivo. **A**–**C** Schematic representation (**A**), tumor growth curves (**B**) and tumor weights (**C**) of the tumor xenografts formed by CDX2- or NUPR1-depleted SW480 cells treated with IKE. **D**–**F** Schematic representation (**D**), tumor growth curves (**E**) and tumor weights (**F**) of the tumor xenografts formed by CDX2- or NUPR1-depleted SW480 cells treated with IKE. **G**, **I**, **K** The levels of intracellular Fe^2+^ (**G**), intracellular MDA (**I**), and PTGS2 mRNA (**K**) in the tumor xenografts formed by CDX2- or NUPR1-depleted SW480 cells. **H**, **J**, **L** The levels of intracellular Fe^2+^ (**H**), intracellular MDA (**J**), and PTGS2 mRNA (**L**) in the tumor xenografts formed by CDX2- or NUPR1-depleted HT-29 cells.

Furthermore, we investigated whether inhibiting the CDX2-NUPR1 pathway could improve the in vivo anticancer efficacy of IKE, a ferroptosis inducer. The tumor xenograft formed by CDX2-knockdown or NUPR1-knockdown HT-29 and SW480 cells were more susceptible to IKE-induced tumor suppression than control groups, which is in line with our in vitro data (Fig. 6A–F). Increased intratumoral Fe^2+^ or MDA levels linked to therapeutic sensitivity was imparted by suppression of the CDX2-NUPR1 pathway (Fig. 6G–J). In addition, the mRNA level of prostaglandin endoperoxide synthase 2 (PTGS2), a marker of ferroptosis, was greatly induced as due to CDX2 or NUPR1 knockdown (Fig. 6K, L). These animal results provide credence to the idea that the CDX2-NUPR1 pathway constrained the anticancer efficacy of IKE.

### Blockage of NUPR1 by ZZW-115 enhancing the anti-cancer efficacy of 5-FU

**Fig. 7:**
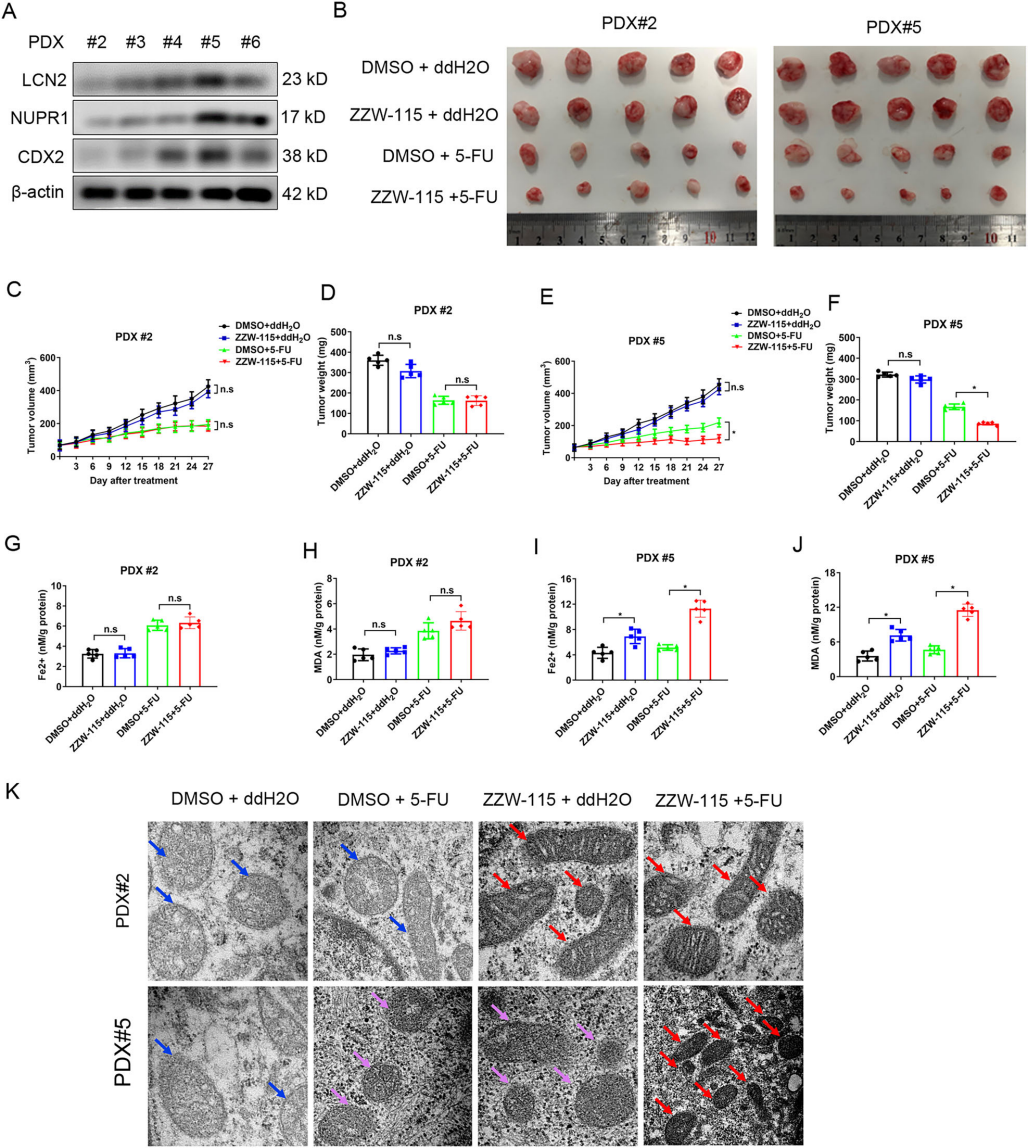
Blockage of NUPR1 by ZZW-115 enhancing the anti-cancer efficacy of 5-FU. **A** Western blotting analysis of CDX2, NUPR1 and LCN2 in tumors generated from four PDX lines of CRC. **B** Schematic representation (**B**) of the tumor xenografts in NSG mice bearing PDX line #2 and #5 treated with 5-FU or ZZW-115 alone or in combination. **C**, **D** The tumor growth curves (**C**) and tumor weights (**D**) of the tumor xenografts s in NSG mice bearing PDX line #2 treated with 5-FU or ZZW-115 alone or in combination. **E**, **F** The tumor growth curves (**E**) and tumor weights (**F**) of the tumor xenografts s in NSG mice bearing PDX line #2 treated with 5-FU or ZZW-115 alone or in combination. **G**, **H** The levels of intracellular Fe^2+^ (**G**) and intracellular MDA (**H**) in the tumor xenografts in NSG mice bearing PDX line #2 treated with 5-FU or ZZW-115 alone or in combination. **I**, **J** The levels of intracellular Fe^2+^ (**I**) and intracellular MDA (**J**) in the tumor xenografts in NSG mice bearing PDX line #5 treated with 5-FU or ZZW-115 alone or in combination. **K** Transmission electron microscopy images of tumor tissues from NSG mice bearing PDX line #2 or #5.

Subsequently, we developed five CRC patient-derived xenograft (PDX) models in NSG mice to determine whether the NUPR1 inhibitor ZZW-115 can increase the therapeutic efficacy of 5-FU [31]. CDX2, NUPR1, and LCN2 were low to moderately expressed in three of these PDX lines (#3, #4, and #6) (Fig. 7A). Out of all the lines, line #2 displayed the lowest levels of these proteins, whereas line #5 displayed the opposite (Fig. 7A). We therefore implanted PDX tumor tissues from lines #2 (the low-CDX2 and NUPR1 level) and #5 (the high-CDX2 and NUPR1 level) into NSG mice for preclinical testing. The mice were divided into four treatment groups: DMSO + ddH_2_O, ZZW-115 + ddH2O, DMSO + 5-FU, and ZZW-115 + 5-FU. Intriguingly, PDX line #2 was more susceptible to 5-FU treatment as compared with PDX line #5 (Fig. 7B–D).

Therapy with ZZW-115 in mice harboring PDX line #2 had no effect on tumor growth either with or without 5-FU co-treatment (Fig. 7B–D). ZZW-115 administration alone showed no impact on tumor growth in mice bearing PDX line #5, however, the combination treatment outperformed 5-FU treatment alone in terms of anti-tumor efficacy (Fig. 7B, E, F). We investigated the occurrence of ferroptosis being linked to the combinatory action on PDX line #2 and #5. ZZW-115 therapy shown a significant synergistic impact with 5-FU on increased intratumoral Fe^2+^ or MDA levels of PDX line #5 but not #2.

Furthermore, tumor cells from the combination treatment group of the PDX line #5 were shown under electron microscopy to have shrunken mitochondria with highly condensed membranes, a morphological sign of ferroptosis (Fig. 7K). Treatment with 5-FU or ZZW-115 alone increased mitochondrial membrane density as well, albeit less so than their combination did (Fig. 7K; the upper panel). The ferroptosis-related morphology of the mitochondria in PDX line #2 was unaffected by ZZW-115 therapy (Fig. 7K), but was significantly raised by 5-FU treatment alone or the combination treatment (Fig. 7k). These findings collectively imply that the NUPR1 inhibitor ZZW-115 improved the ferroptosis-inducing action of 5-FU on colon cancer with highly-expressed of CDX2 and NURP1.

## Discussion

Recent research has shown that CDX2 expression is a reliable predictor of favorable survival in CRC [32–34]. Paradoxically, CDX2 is associated with lower response rates to systemic chemotherapy in stage II-III CRC [8]. Herein, we directly showed by employing in vitro and in vivo models that CDX2 confers chemotherapeutic resistance in stage II-III colon cancer. Consistently, recent research by Bruun et al. has revealed the proposed predictive value of CDX2 for adjuvant chemotherapy in stage III CRC [35]. However, additional publications and data did not support the potential value of CDX2 in predicting chemotherapy effectiveness. A meta-analysis conducted that CDX2 expression was not related to the efficiency of chemotherapy in stage II-III solid tumors including CRC [36]. Ryan et al. showed that there was no difference in response to chemotherapy based on CDX2 expression in stage II-III dMMR CRC. This discrepancy might be attributed to sample sizes, therapy regimen, and clinicopathological variables, including MSI status, BRAF mutations, CMS, and TNM stage [37, 38].

Notably, regarding stage IV CRC, the value of CDX2 on predicting chemotherapeutic benefit remain still in disputes. A study revealed that CDX2 abundance improved the response to adjuvant treatment in solid tumors including TNM IV stage CRC [36]. Conversely, Bruun et al. reported that no indication of an association between CDX2 expression and benefit from chemotherapy was observed in stage IV CRC [35]. Hence, the predictive utility of CDX2 can vary on the tumor stage.

Apoptosis induction with anti-cancer therapy has historically been one of the main strategies for eliminating cancer cells [39]. However, due to the innate or acquired resistance to apoptosis, apoptosis induction in tumors is only partially effective [40]. Therefore, taking advantage of non-apoptotic cell death in alternative ways creates new therapeutic opportunities for eradicating cancer cells and reducing the spread of drug-resistant clones. In comparison to normal, non-cancer cells, cancer cells have a higher requirement for iron [41]. Because of their iron dependency, cancer cells may be more susceptible to ferroptosis induction, which elicits cell death in chemotherapy-resistant cancer cells. Nevertheless, heterogeneity in a tumor allows a small portion of cancer cells to survive and adopt distinct defense mechanisms in blocking ferroptosis, which ultimately leads to drug resistance and cancer relapse [42]. Herein, we show for the first time that CDX2 reduction increases ferroptotic cell death brought on by erastin or RSL3 as well as cell viability loss and ROS production. The CDX2 depletion-induced cell death was reversed by ferroptosis rescue drugs but not by apoptosis inhibitor or necroptosis inhibitor. We successfully engineered ferroptosis resistance in colon cancer cells that overexpress CDX2. These results show that ferroptosis is negatively regulated by CDX2.

Ferroptosis resistance induced by the NUPR1-LCN2 pathway has drawn considerable attention [23]. As the direct target of NUPR1, LCN2 contributes to iron metabolism through extruding iron out of cells and into the extracellular space [43, 44]. For the first time, the current research demonstrated that CDX2 directly controls the function of NUPR1 as a downstream target gene. The increased intracellular iron concentrations and oxidative damage, including lipid peroxidation and DNA damage, brought about by CDX2 depletion were eliminated by increasing NUPR1 expression, whereas the ferroptosis resistance brought about by CDX2 overexpression was countered by silencing NUPR1 expression.

Coincidentally, Hinoi et al. found that CDX2 induction suppressed intracellular iron levels via transactivating hephaestin to regulate iron export [45]. According to our research, CDX2 induces NUPR1 expression, which prevents ferroptotic cell death. In response to numerous stimuli, including ER stress, ATF4, a repressor of ferroptosis, regulates NUPR1 expression [24, 46]. A recent study by Liu et al. further confirmed that ATF4 is essential for NUPR1 induction upon ferroptosis agonists [23]. Future research needs to clarify whether ATF4 is involved in CDX2-mediated NUPR1 expression.

Our results point to a potential functional role for the CDX2-NUPR1 pathway in the control of tumor therapy for ferroptosis induction. By in vitro or in preclinical mice models, we discovered that anticancer efficacy of ferroptosis agonists in colon cancer was improved by the pharmacological or genetic suppression of the CDX2-NUPR1 pathway. Ferroptosis appears to have a dual function in the biology of tumors dependent on specific environment [47, 48]. Ferroptosis activators can eradicate cancer cells or stimulate active antitumor immunity to halt the tumor growth. However, the inflammatory reaction brought on by ferroptosis may accelerate the growth of tumors by releasing DAMPs [49, 50]. To improve anticancer therapy approaches, more investigation of the cellular and functional significance of ferroptosis in tumor microenvironment is required.

The concept that CDX2, a gene that inhibits carcinogenesis, would be connected to chemoresistance seems paradoxical. It is possible to contend that CDX2 serves both an oncogene and a tumor suppressor gene role. In fact, CDX2 reduces intestinal carcinogenesis, as has been conclusively demonstrated by us and others [51–54], but CDX2 also serves as an oncogene in a number of other malignancies, including leukemia, esophageal, and gastric cancers [55–57]. Although the exact nature of this adaptability is still unclear, it may be influenced by the tumor microenvironment and molecular context [58]. Another hypothesis is that colon cancer cells require the biological properties of CDX2 for their own survival. In fact, CDX2 is an intestine-specific transcription factor essential for the physiological function of intestine [59]. Colon epithelium, serving as a barrier to the lumen’s content, adopt a defensive mechanism, particularly against xenobiotics. The activation of NUPR1 by CDX2 might be a protective mechanism underlying the reaction of intestinal mucosa to the external stimulus to maintain intestinal homeostasis.

The current study demonstrated a positive correlation between CDX2 expression and chemical resistance in colon cancer. Mechanistically, CDX2 directly transactivates NUPR1 and subsequent its target LCN2 to confer ferroptosis resistance by inhibiting iron-induced oxidative damage. Genetic or pharmacological blockage of CDX2-NUPR1 axis may strengthen the anticancer efficacy of adjuvant chemotherapy on stage II-III CDX2-positive colon cancer in vitro and in vivo. Our study uncovered a novel molecular mechanism by which CDX2 confers ferroptosis resistance in colon cancer. Blockage of NUPR1 might be as a potential therapeutic strategy for stage II-III CDX2-positive colon cancer.

However, our study still has several limitations, which impacts the interpretation of results. The biological test and mechanism assays we mostly conducted is at the in vitro level and could not yet reflect the in vivo information of CDX2 in the chemical resistance. Moreover, patients with II-III colon cancer mostly receive the combination chemotherapy regimen. The real-world setting of chemical response is quite complicated. For metastatic CRC (mCRC), the FOLFOXIRI plus cetuximab or bevacizumab is proposed as the first-line treatment [60]. The present study did not evaluate the response to irinotecan or monoclonal antibody. The conclusion we draw could not extend to mCRC.

## Supplementary information

Uncropped western blot

Supplementary figure 1

Supplementary figure 2

Supplementary figure 3

Supplementary figure 4

Supplementary legend

Supplementary table

## Data Availability

All data needed to evaluate the conclusions in the paper are present in the paper and/or the Supplementary Materials. Additional data related to this paper may be requested from the authors.
