# Supplementary figures and images for: CDX2 confers ferroptosis resistance in stage II-III colon cancer via upregulation of NUPR1
Source: Cell Death Dis. 2026 Mar 12;17(1):308. doi: 10.1038/s41419-026-08412-x (PMC13039209; doi:10.1038/s41419-026-08412-x)

Fig.2D

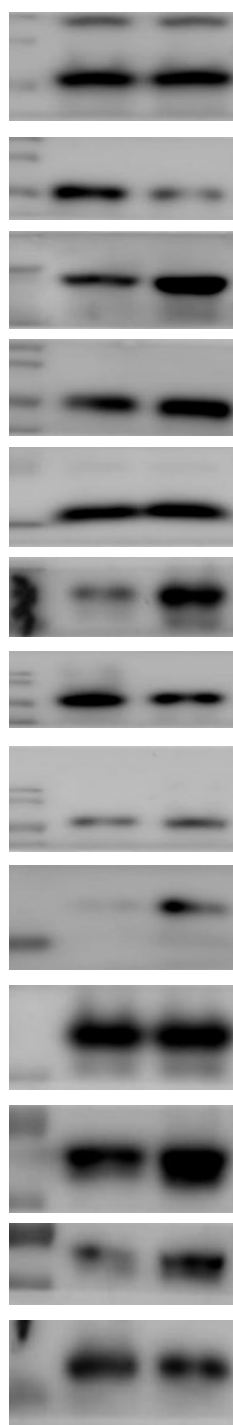

Fig.2E

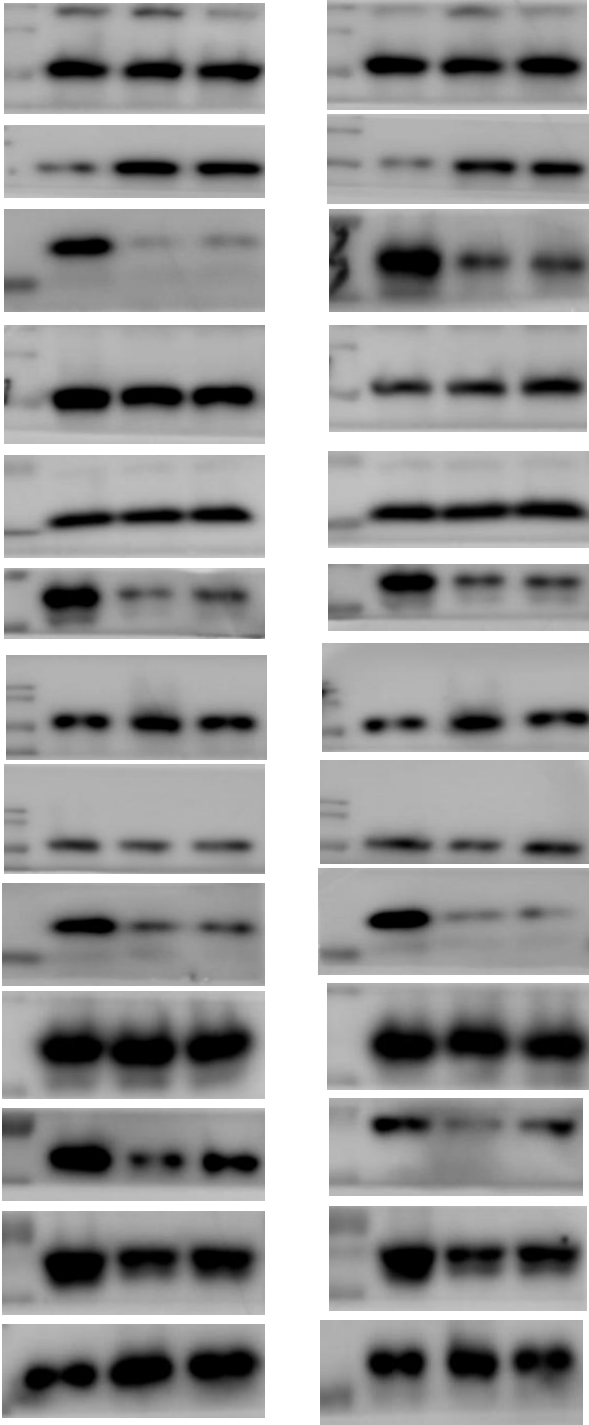

Fig.5B

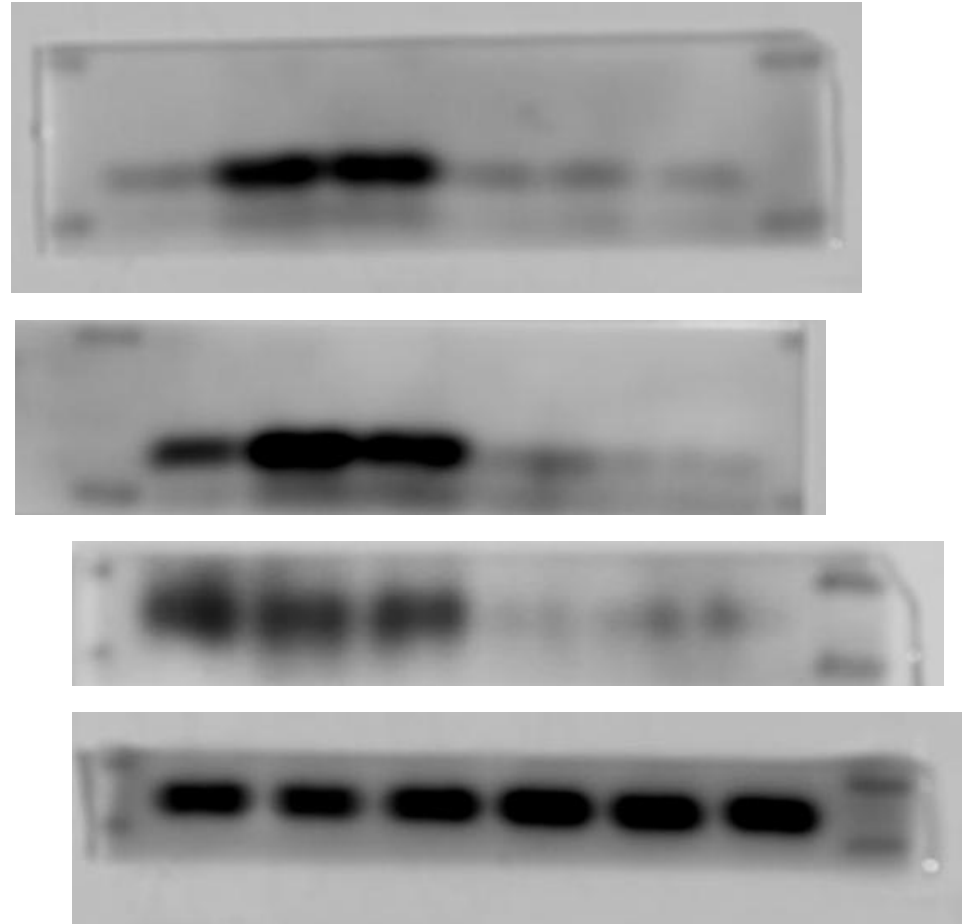

Fig.5C

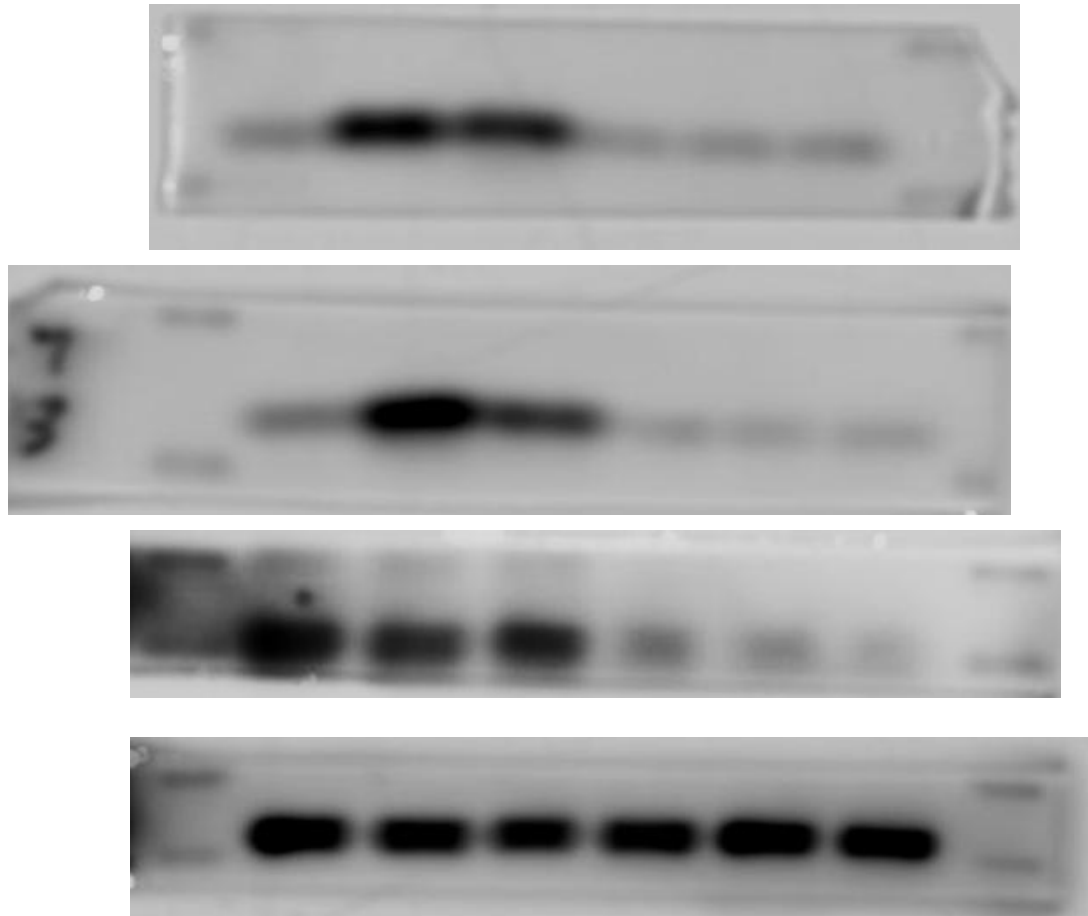

Fig.7A

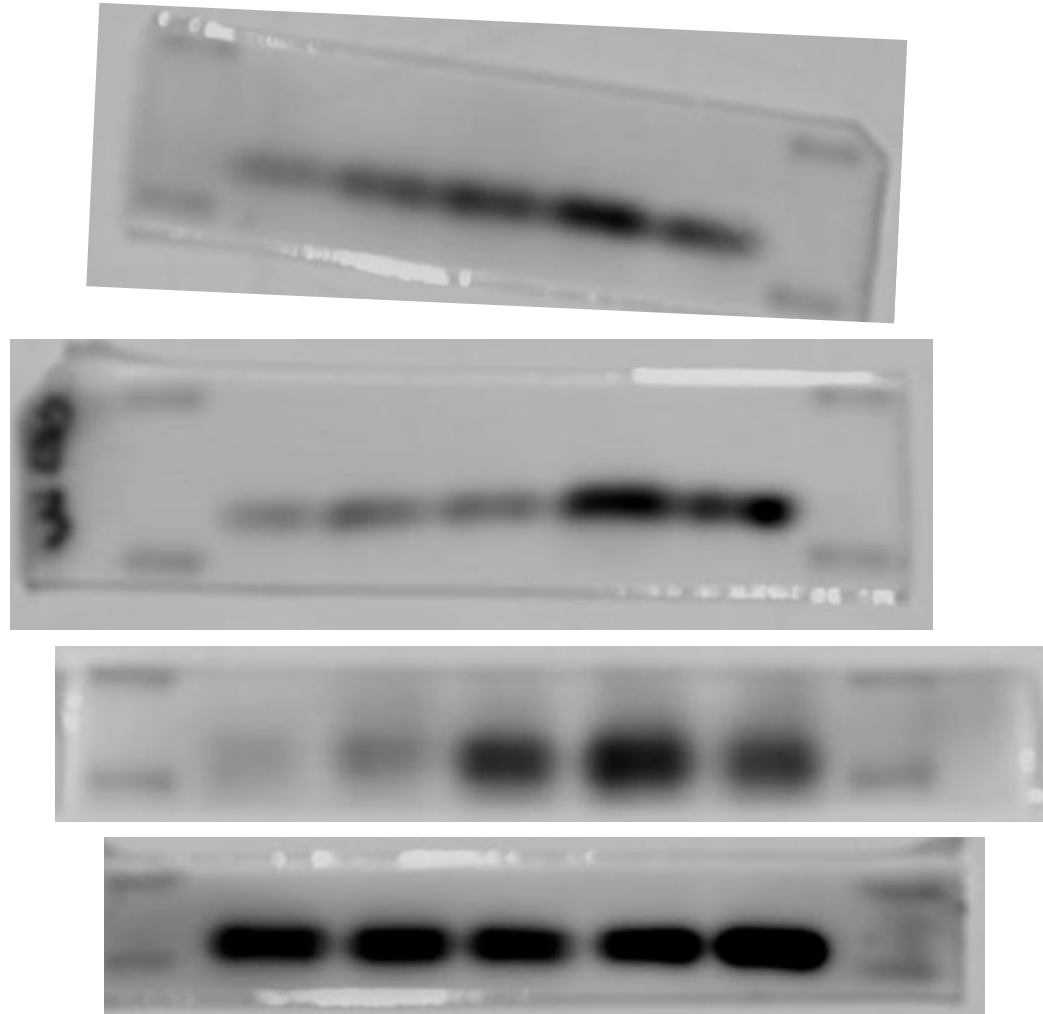

Supplemental Fig.4B

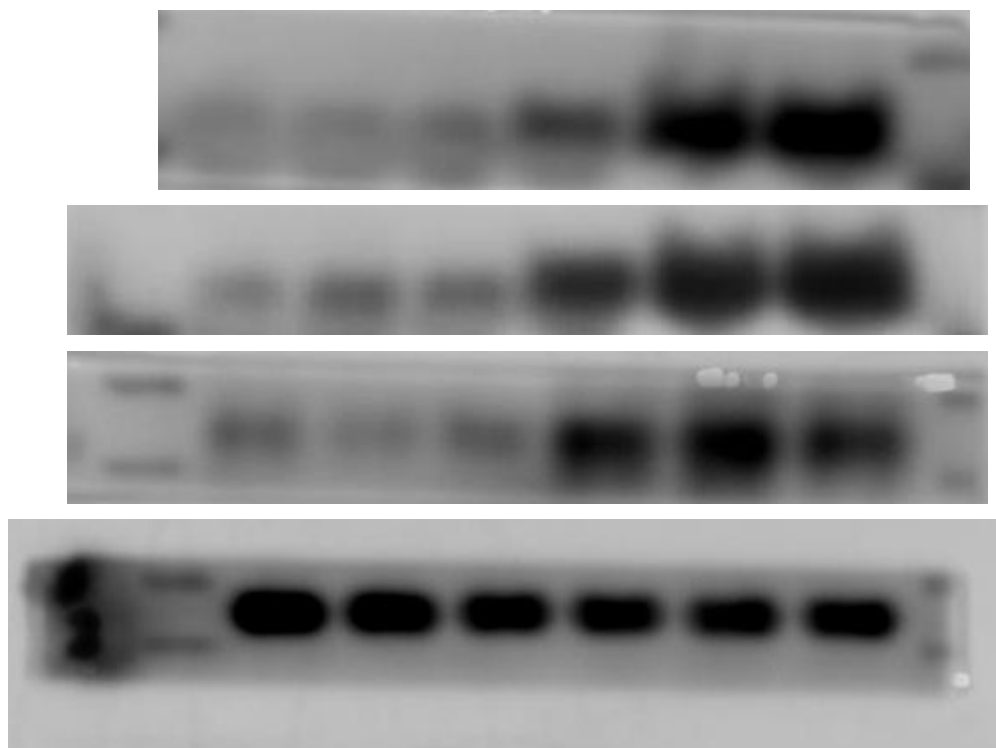

Supplement: Supplementary file 1 — Uncropped western blot [file 41419_2026_8412_MOESM1_ESM.pdf]

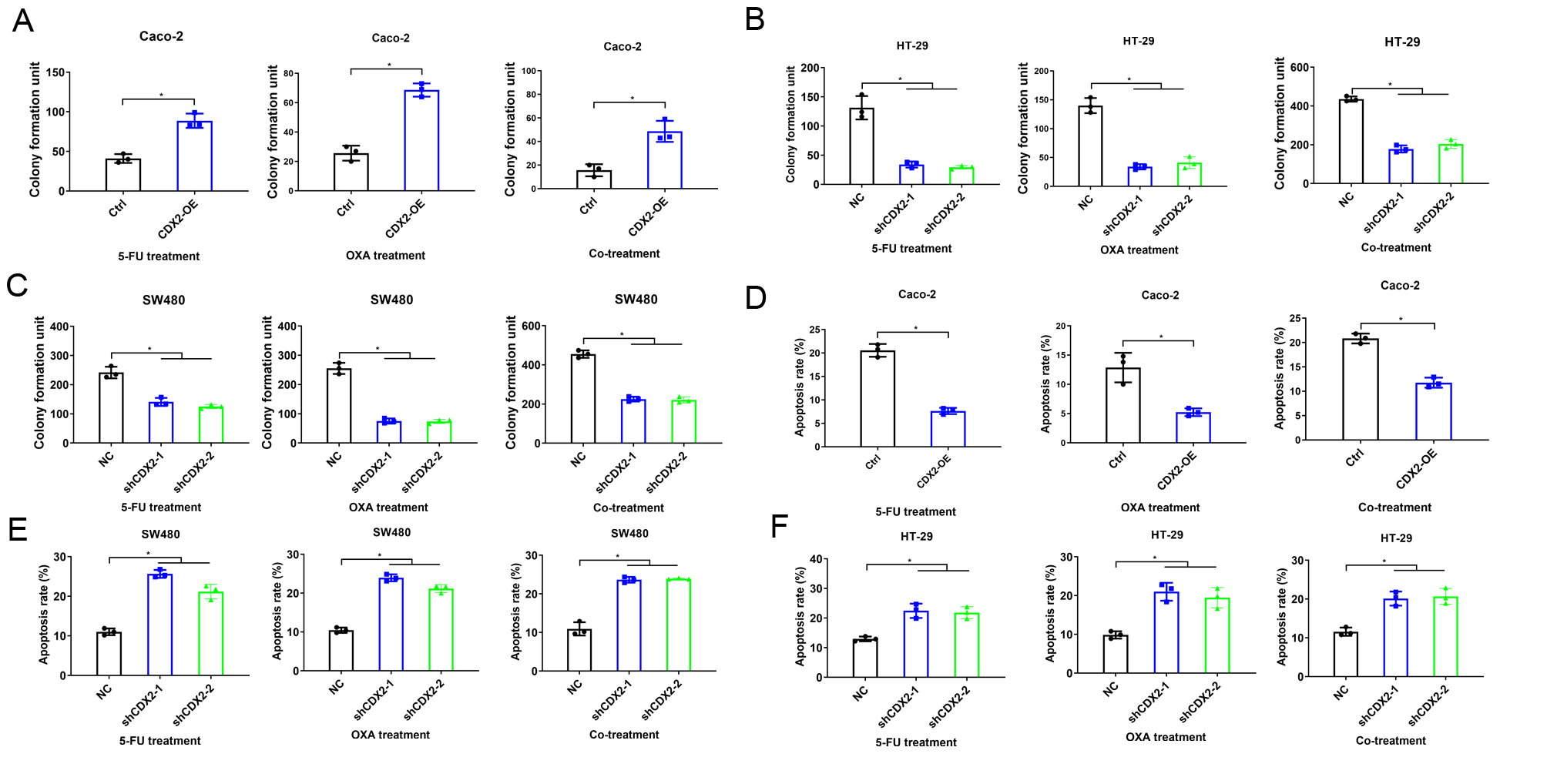

Supplement: Supplementary file 2 — Supplementary figure 1 [file 41419_2026_8412_MOESM2_ESM.tif]

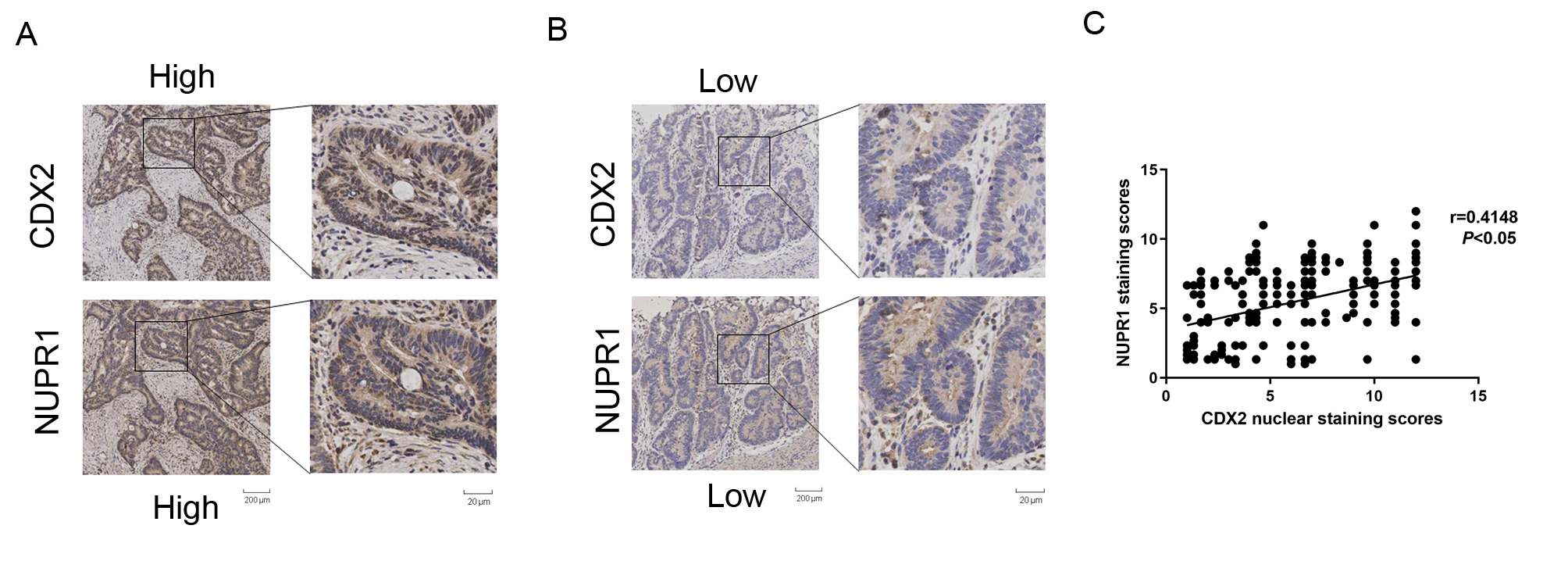

Supplement: Supplementary file 3 — Supplementary figure 2 [file 41419_2026_8412_MOESM3_ESM.tif]

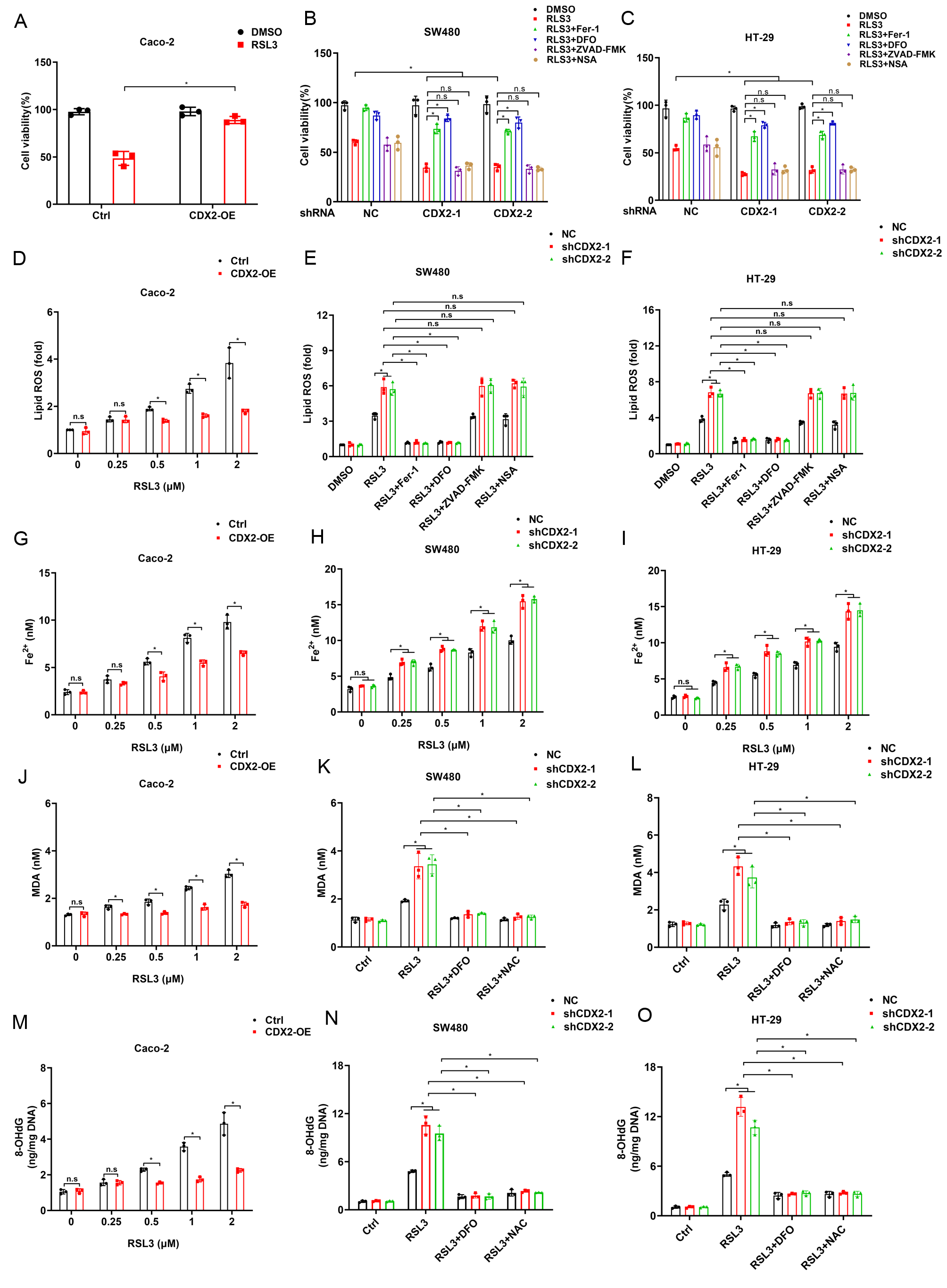

Supplement: Supplementary file 4 — Supplementary figure 3 [file 41419_2026_8412_MOESM4_ESM.tif]

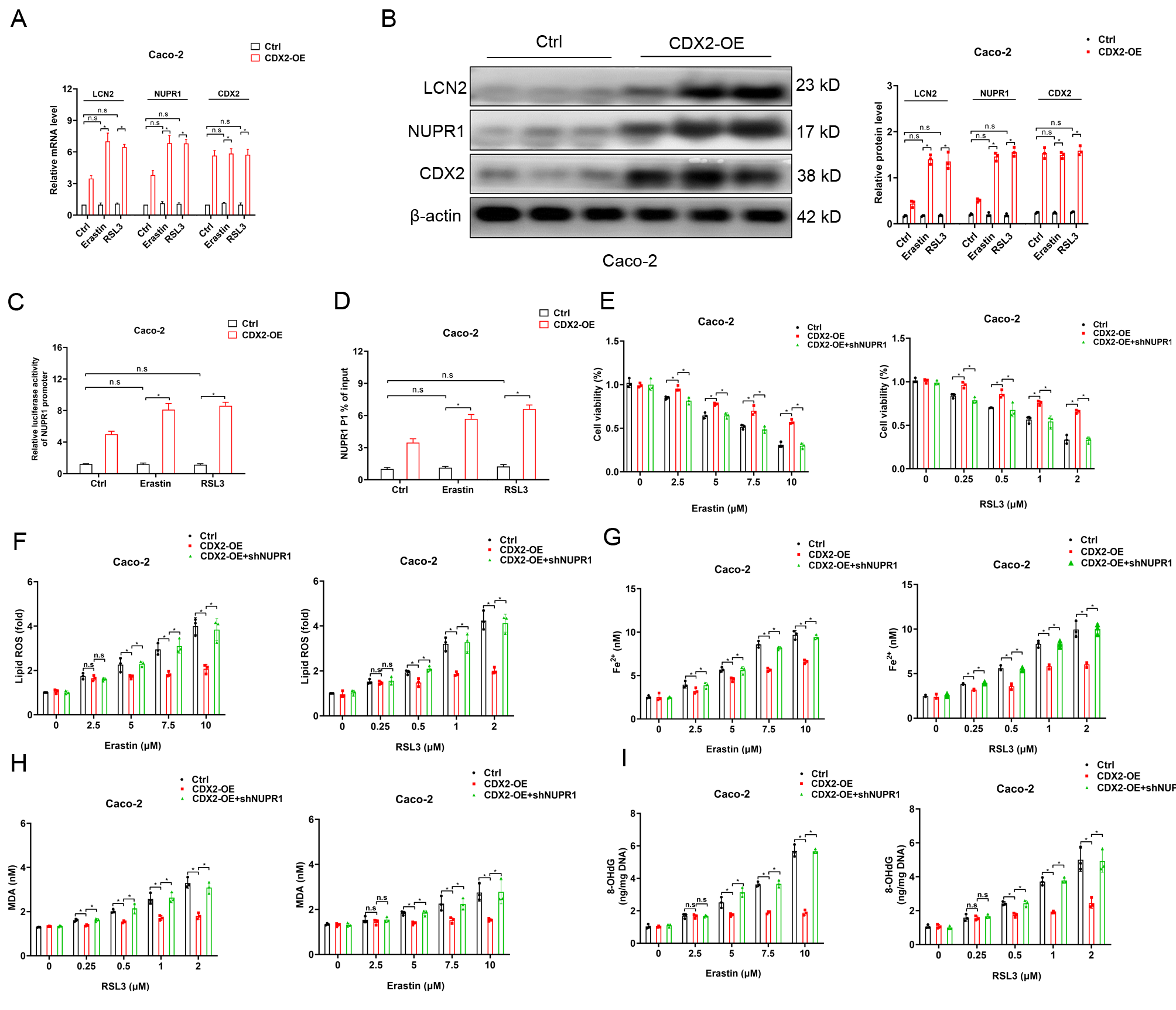

Supplement: Supplementary file 5 — Supplementary figure 4 [file 41419_2026_8412_MOESM5_ESM.tif]
