# Supplementary material for: CDX2 confers ferroptosis resistance in stage II-III colon cancer via upregulation of NUPR1
Source: Cell Death Dis. 2026 Mar 12;17(1):308. doi: 10.1038/s41419-026-08412-x (PMC13039209; doi:10.1038/s41419-026-08412-x)
Supplement: Supplementary file 6 — Supplementary legend [file 41419_2026_8412_MOESM6_ESM.docx]

**Figure legend**

**Fig. 1 CDX2 promoted the chemical resistance of colon cancer** **a** Comparison of colony formation of CDX2-overexpressing Caco-2 cells and the control cells to 5-FU or oxaliplatin alone or in combination. **b**, **c** Comparison of colony formation of CDX2-depleted SW480 (b) and HT-29 (c) cells and the control cells to 5-FU or oxaliplatin alone or in combination. **d** Comparison of apoptosis of CDX2-overexpressing Caco-2 cells and the control cells to 5-FU or oxaliplatin alone or in combination. **e**, **f** Comparison of apoptosis of CDX2-depleted SW480 (**e**) and HT-29 (**f**) cells and the control cells to 5-FU or oxaliplatin alone or in combination. All data are the mean ± SD of three independent experiments. **P* < 0.05.

**Fig. 2 NUPR1 expression is positively correlated with CDX2 in Stage II-III Colon Cancer** CDX2 and NUPR1 expression (a, high; b, low ) in an independent cohort II of 228 stage II-III colon cancer tissues from CRC patients underwent surgery at the First Affiliated Hospital of Xi’an Jiaotong University. c Correlation analysis of CDX2 and NUPR1 in CRC tissue microarrays. **P* < 0.05

**Fig. 3 CDX2 protects colon cancer cells from ferroptosis through the inhibition of iron-dependent oxidative damage a**, **d**, **g**, **j**, **m** The effect of CDX2 overexpression on cell viability (**a**), lipid ROS (**d**), intracellular Fe^2+^ (**g**), intracellular MDA (**j**), and intracellular 8-OHdG (**m**) of Caco-2 cells to RSL3. **b**, **e**, **h**, **k**, **n** The effect of CDX2 depletion on cell viability (**b**), lipid ROS (**e**), intracellular Fe^2+^ (**h**), intracellular MDA (**k**), and intracellular 8-OHdG (**n**) of SW480 cells to RSL3 in the absence or presence of Fer-1, DFO, ZVAD-FMK, or NSA. **c**, **f**, **i**, **l**, **o** The effect of CDX2 depletion on cell viability (**c**), lipid ROS (**f**), intracellular Fe^2+^ (**i**), intracellular MDA (**l**), and intracellular 8-OHdG (**o**) of HT-29 cells to RSL3 in the absence or presence of Fer-1, DFO, ZVAD-FMK, or NSA. All data are presented as the mean ± SD from three independent experiments. **P* < 0.05.

**Fig. 4 NUPR1 acts as an effector gene of CDX2 in blocking ferroptosis a** The mRNA levels of CDX2, NUPR1 and LCN2 in CDX2-overexpressing Caco-2 cells exposed to erastin and RSL3 detected by real-time PCR. **b** The protein levels of CDX2, NUPR1, and LCN2 in CDX2-overexpressing Caco-2 cells cells exposed to erastin and RSL3 detected by western blotting analysis. **c** The activities of the NUPR1 full promoter reporter construct in CDX2-overexpressing Caco-2 cells exposed to erastin and RSL3 using the dual-luciferase assay. **d** Enrichment level of the CDX2-binding putative site (P1) in the NUPR1 promoter region in CDX2-overexpressing Caco-2 cells exposed to erastin and RSL3 determined by the qChIP assay. **e**, **f**, **g**, **h**, **i** The effect of NUPR1 depletion on cell viability (**e**), lipid ROS (**f**), intracellular Fe^2+^ (**g**), intracellular MDA (**h**), and intracellular 8-OHdG (**i**) of CDX2-overexpressing Caco-2 cells exposed to erastin and RSL3. All data are presented as the mean ± SD from three independent experiments. **P* < 0.05.
