# Supplementary material for: CDX2 confers ferroptosis resistance in stage II-III colon cancer via upregulation of NUPR1
Source: Cell Death Dis. 2026 Mar 12;17(1):308. doi: 10.1038/s41419-026-08412-x (PMC13039209; doi:10.1038/s41419-026-08412-x)
Supplement: Supplementary file 7 — Supplementary table [file 41419_2026_8412_MOESM7_ESM.docx]

Supplementary Table 1 Primer sequence

| Gene | Sequence | |
| --- | --- | --- |
| RT-PCR | | |
| CDX2 | | F: 5’-GAACCTGTGCGAGTGGATG-3’ |
|  | | R: 5’-GGATGGTGATGTAGCGACTG-3’ |
| NUPR1 | | F: 5’-GACTCCAGCCTGGATGAATCTG-3’ |
|  | | R: 5’-CTTCTCTCTTGGTGCGACCTTTC-3’ |
| LCN2 | | F: 5’-GTGAGCACCAACTACAACCAGC-3’ |
|  | | R: 5’-GTTCCGAAGTCAGCTCCTTGGT-3’ |
| PTGS2 | | F: 5’-CGGTGAAACTCTGGCTAGACAG-3’ |
|  | | R: 5’-GCAAACCGTAGATGCTCAGGGA-3’ |
| GAPDH | | F: 5’-TGCACCACCAACTGCTTAGC-3’ |
|  | | R: 5’-GGCATGGACTGTGGTCATGAG-3’ |
| Luciferase Assays | |  |
| F1(-2931 bp **-** +174 bp) | | F: 5’-TGAGTGCCAGGCATTGAGGATACA-3’ |
| F2(-2566 bp **-** +174 bp) | | F: 5’-CGGCCTCCCAAAGTGCTGGG-3’ |
| F3(-1838 bp **-** +174 bp) | | F: 5’-TGCCCAAGCTGGCCTTGAACT-3’ |
| F4(-929 bp **-** +174 bp) | | F: 5’-ACCCTCTGCCACCCTCCTCC-3’ |
|  | | R: 5’-AGGGGTGGGTCCTGATTTCGG-3’ |
| ChIP-qPCR  P1(-3010 bp **-** -2751 bp) | | F: 5’-GCAGGTGGAAGCTGAGCAAGCA-3’ |
|  | | R: 5’-GATCGCGCCACTGCACTCCA-3’ |
| P2(-2280 bp **-** -2080 bp) | | F: 5’-AGACCTGAGAAGCCCTGTTCCAGA-3’ |
|  | | R: 5’-GGCTGGTCTCGAACTCCTGACCTC-3’ |
| P3(-2141 bp **-** -1938 bp) | | F: 5’-CCCAGCACTTTGGGAGGCCG-3’ |
|  | | R: 5’-GGCTCACTGCAACCTCCGCC-3’ |
| P4(-1270 bp **-** -1071 bp) | | F: 5’- CCCAAAGTGCTGGGACTACAGGC-3 |
|  | | R: 5’-TTCTTCCTAGAGTTGGGGAGAGGATCA -3’ |
| 3’-UTR | | F: 5’-TCCTGGCCTCTGATTGGCCT-3’ |
|  | | F: 5’-AAGCCCCTGGGGTAGGACAG-3’ |

Supplemental Table 2 Antibodies

| Antibodies | Source | | Identifier |
| --- | --- | --- | --- |
| β-actin | | Abcam | Cat#ab8226 |
| CDX2 | | Cell Signaling Technology | Cat#12306S |
| NUPR1 | | Abcam | Cat#ab234696/6028 |
| LCN2 | | Cell Signaling Technology | Cat#44058 |
| ACSL3 | | Abcam | Cat#ab151959 |
| ACSL4 | | Abcam | Cat#ab155282 |
| AKR1C1 | | Abcam | Cat#ab192785 |
| ALOX15 | | Abcam | Cat#ab244205 |
| GPX4 | | Abcam | Cat#ab125066 |
| NCOA4 | | Abcam | Cat#ab86707 |
| NFE2L2 | | Abcam | Cat#ab62352 |
| NQO-1 | | Abcam | Cat#ab80588 |
| SLC1A5 | | Abcam | Cat#ab237704 |
| SLC7A11 | | Abcam | Cat#ab175186 |
